# Lytic and mechanical stability of clots composed of fibrin and blood vessel wall components

**DOI:** 10.1111/jth.12112

**Published:** 2013-03-13

**Authors:** Z Rottenberger, E Komorowicz, L Szabó, A Bóta, Z Varga, R Machovich, C Longstaff, K Kolev

**Affiliations:** * Department of Medical Biochemistry, Semmelweis UniversityHungary; † Institute of Materials and Environmental Chemistry, Research Centre for Natural Sciences, Hungarian Academy of SciencesHungary; ‡ Department of Biological Nanochemsitry, Institute of Molecular Pharmacology, Research Centre for Natural Sciences, Hungarian Academy of SciencesBudapest, Hungary; § Biotherapeutics, Haemostasis Section, National Institute for Biological Standards and ControlSouth Mimms, Potters Bar, UK

**Keywords:** decorin, fibrin, glycosaminoglycan, plasmin, rheology, shear stress

## Abstract

**Background:**

Proteases expressed in atherosclerotic plaque lesions generate collagen fragments, release glycosaminoglycans (chondroitin sulfate [CS] and dermatan sulfate [DS]) and expose extracellular matrix (ECM) proteins (e.g. decorin) at sites of fibrin formation.

**Objective:**

Here we address the effect of these vessel wall components on the lysis of fibrin by the tissue plasminogen activator (tPA)/plasminogen system and on the mechanical stability of clots.

**Methods and results:**

MMP-8-digested collagen fragments, isolated CS, DS, glycosylated decorin and its core protein were used to prepare mixed matrices with fibrin (additives present at a 50-fold lower mass concentration than fibrinogen). Scanning electron microscopy (SEM) showed that the presence of ECM components resulted in a coarse fibrin structure, most pronounced for glycosylated decorin causing an increase in the median fiber diameter from 85 to 187 nm. Rheological measurements indicated that these structural alterations were coupled to decreased shear resistance (1.8-fold lower shear stress needed for gel/fluid transition of the clots containing glycosylated decorin) and rigidity (reduction of the storage modulus from 54.3 to 33.2 Pa). The lytic susceptibility of the modified fibrin structures was increased. The time to 50% lysis by plasmin was reduced approximately 2-fold for all investigated ECM components (apart from the core protein of decorin which produced a moderate reduction of the lysis time by 25%), whereas fibrin-dependent plasminogen activation by tPA was inhibited by up to 30%.

**Conclusion:**

ECM components compromise the chemical and mechanical stability of fibrin as a result of changes in its ultrastructure.

## Introduction

Fatal outcomes in ischemic cardio- and cerebrovascular disease are not necessarily a consequence of critical narrowing of the atherosclerotic artery; rather rupture or erosion of the plaque provokes formation of unstable thrombi, the detachment of which can occlude downstream branches of the vascular tree (reviewed in 1). Convincing histopathological data support the notion that such microembolization is the ultimate cause of myonecrosis in patients dying of acute coronary thrombosis 2. Although the mechanisms triggering thrombus formation on atherosclerotic plaques are well characterized, little is known about the factors that determine the stability of these thrombi and thus their capacity to form emboli. Because the solid matrix of thrombi is composed of fibrin that originates from a blood-borne protein (fibrinogen), the molecular events at the interface of blood and damaged vascular wall appear to be crucial with respect to this stability. A major role in plaque disruption is attributed to matrix metalloproteinases (MMP) expressed in the vessel wall (reviewed in 3). Infiltration of thrombi by leukocytes supplies additional MMPs and serine proteases (e.g. neutrophil elastase) at this interface (reviewed in 4). We have recently demonstrated that such cell- (MMP8, MMP9, elastase) and thrombus-related (thrombin, plasmin) proteolytic activity results in clearance of the glycosaminoglycans (GAGs) and modification of the extracellular matrix protein (ECM) structure in the arterial wall 5. Based on these findings, we hypothesized that vascular wall components released through proteolysis could modify the stability of the fibrin matrix formed at the thrombus/vessel wall interface. Both mechanical stability and susceptibility to the action of the tissue plasminogen activator (tPA)/plasminogen system may be affected (reviewed in 6). In the present study we addressed the stability of fibrin matrices co-polymerized *in vitro* with vascular proteins, or their fragments, and GAG side chains, selected either because of their abundance (type I and III collagens are the major ECM proteins of the arteries 7,8) or known effects in blood coagulation and fibrinolysis (decorin 9,10, chondroitin sulfate and dermatan sulfate 11).

## Materials and methods

Human fibrinogen (plasminogen free) was the product of Calbiochem (La Jolla, CA, USA). This preparation contained low levels of contaminating factor (F)XIII, the activity of which resulted in depletion of γ-chain monomers in reducing SDS polyacrylamide electrophoresis within 1 h after clotting at the thrombin and CaCl_2_ concentrations used in these experiments. In the absence of CaCl_2_ (in the rheological assay), no γ-dimers could be detected within 30 min. The chromogenic substrates for plasmin, Spectrozyme-PL (H-D norleucyl-hexahydrotyrosyl-lysine-*p*-nitroanilide) and for thrombin, Spectrozyme-TH (H-D-hexahydrotyrosyl-L-alanyl-L-arginine-*p*-nitroanilide) were from American Diagnostica (Pfungstadt, Germany), and tPA was from Boehringer Ingelheim (Germany). Bovine thrombin was purchased from Serva (Heidelberg, Germany) and was further purified by ion-exchange chromatography on sulfopropyl-Sephadex yielding a preparation with a specific activity of 2100 IU mg^−1^ 12. Dermatan sulfate and chondroitin sulfate were from Sigma-Aldrich Kft. (Budapest, Hungary). Collagen G was from Biochrom AG (Berlin, Germany). Recombinant human decorin core protein was a product of R&D Systems (Abingdon, England). Blood was collected from healthy volunteers with venipuncture in 10 mm trisodium citrate (final concentration) and after a 10-min centrifugation at 2000 × *g* the top three-quarters of the plasma layer was used for the measurements within 4 h.

### Purification of fully glycosylated decorin

Published protocols were used for the purification of full-length glycosylated decorin (aorta decorin) 13, with modifications. Freshly cut bovine aorta slices were immediately frozen and stored at −80 °C in 2-methylbutane. Thawed aorta pieces were homogenized in 4 m guanidine hydrochloride, 50 mm sodium acetate pH 5.6 buffer containing 5 mm benzamidine, 100 mm ε-aminocaproic acid, 10 mm EDTA 1 mm phenylmethanesulfonyl fluoride (10 mL extraction buffer g^−1^ tissue). After an overnight incubation, the extract was centrifuged at 4 °C, 10 000 × *g* for 1 h and the supernatant was dialyzed at 4 °C overnight against 50 mm sodium-acetate pH 6.0 buffer containing 8 m urea, 200 mm NaCl, 0.1% Triton-X-100 (buffer D). The dialyzed extract was batch adsorbed with buffer D equilibrated Q-Sepharose and stirred for 2 h at 4 °C. A Q-Sepharose column was washed with five column volumes of buffer D, and step elution performed with 0.5–1.2 m NaCl in buffer D. Fractions were analyzed with alcian-blue staining after electrophoresis in polyacrylamide gel (SDS-PAGE). Proteoglycan-containing fractions were pooled and dialyzed overnight at 4 °C against 50 mm sodium acetate pH 6.0 buffer containing 7 m urea, 100 mm NaCl (buffer D2). A DEAE-Sepharose column was equilibrated with 10 volumes of buffer D2 and mixed with the dialyzed fractions. After washing the column with 5 column volumes of D2, gradient elution was carried out with 0.3–1.2 m NaCl in D2. Glycosaminoglycan containing fractions were pooled and dialyzed against 50 mm sodium acetate pH 6.0 buffer containing 1.5 m ammonium-sulfate (buffer D3) overnight at 4 °C. An Octyl-Sepharose column was washed with 10 column volumes of buffer D3, mixed with the proteoglycan-containing dialyzed fractions, washed with 5 column volumes of buffer D3 and eluted with a 1.5–0 m ammonium-sulfate gradient. Fractions were analyzed after SDS-PAGE with alcian blue staining and anti-decorin antibody (Santa Cruz Biotechnology Inc., Santa Cruz, CA, USA). Decorin-containing fractions were dialyzed in 10 mm HEPES pH 7.4 buffer containing 150 mm NaCl and stored at −20 °C. Decorin was verified with mass spectrometric analysis of its tryptic peptides on LCQ-Fleet Ion Trap mass spectrometer (Thermo Scientific, Karlsruhe, Germany).

### Generation of collagen fragments

Recombinant MMP-8 (R&D Systems) at 1 mg mL^−1^ in 50 mm HEPES buffer pH 7.5 containing 150 mm NaCl, 1 mm CaCl_2_ was activated with 1 mm aminophenylmercuric-acetate (APMA) at 37 °C for 1 h. Collagen G (90:10% mixture of collagen I and III) was digested with the activated enzyme at 37 °C for 8 h (1 mg collagen in 1 mL reaction volume in the same buffer). The process was stopped with 30 μL 50 mm HEPES, 45 mm EGTA pH 8.0. This digestion resulted in collagen fragments in the range of 20–60 kDa as verified with silver staining after SDS-PAGE.

### Turbidimetric fibrinolytic assays

In 96-well microtiter plates, 6 μm fibrinogen in 10 mm HEPES buffer pH 7.4 containing 150 mm NaCl, 2 mm CaCl_2_ and collagen fragments/decorin core protein/aorta decorin/dermatan sulfate/chondroitin sulfate were mixed with 20 nm thrombin and 6 nm plasmin in a total volume of 100 μL. Clot formation and dissolution was followed by measuring the light absorbance at 340 nm at 37 °C with a Zenyth 200rt microplate spectrophotometer. The lysis time (*t*_*1/2*_), defined as the time needed to reduce the turbidity of the clot to a half-maximal value, was used as a quantitative parameter of fibrinolytic activity. In a preliminary series of experiments a range of additive concentrations between 1 and 200 μg mL^−1^ was screened in this assay and the minimal concentrations yielding significant differences in the lysis time were selected for more detailed investigation (2 μg mL^−1^ for the two forms of decorin and 50 μg mL^−1^ for the rest of the additives). In certain cases, plasma containing 50 ng mL^−1^ tPA was clotted with 15 nm thrombin and 12.5 mm CaCl_2_.

### Scanning electron microscopic studies

Fibrin clots of 50 μL volume were prepared in duplicate: 6 μm fibrinogen in 10 mm HEPES buffer pH 7.4 containing 150 mm NaCl and 2 mm CaCl_2_ was clotted with 20 nm thrombin at 37 °C for 1 h, with or without vessel wall proteins/glycosaminoglycans at final concentrations 50 μg mL^−1^ for collagen fragments, dermatan sulfate or chondroitin sulfate and 50 nm for decorin core protein or aorta decorin. Clots were washed repeatedly with 0.5% Triton X-100 and placed in 100 mm Na-cacodylate pH 7.2 for 1 h. After fixation in 1% glutaraldehyde for 1 h, the samples were dehydrated in a series of ethanol dilutions (20–50–70–85–96%, 5 min each), 1:1 mixture of 96% ethanol:acetone and finally with pure acetone, followed by critical point drying with CO_2_ in an E3000 Critical Point Drying Apparatus (Quorum Technologies, Newhaven, UK). The samples were mounted on adhesive carbon discs, sputter coated with gold in SC7620 Sputter Coater (Quorum Technologies) and images were taken with scanning electron microscope EVO40 (Carl Zeiss GmbH, Oberkochen, Germany). The scanning electron microscopic (SEM) images were analyzed to determine the diameter of the fibrin fibers using self-designed program functions running under the Image Processing Toolbox v. 8.0 of Matlab 7.14.0.739 (R2012a) (The Mathworks, Natick, MA) as previously described 14.

### Plasminogen activation assay

In 96-well microtiter plates, 3.3 μm fibrinogen in 10 mm HEPES buffer pH 7.4 containing 150 mm NaCl, 1.5 mm CaCl_2_, 75 nm plasminogen and vessel wall proteins or glycosaminoglycans (at concentrations given above for the SEM samples) was clotted with 25 nm thrombin in a volume of 80 μL. After 45 min at 37 °C, 60 μL of 14 nm tPA and 0.6 mm Spectrozyme-PL in 10 mm HEPES, 150 mm NaCl pH 7.4 were placed on the surface of the clot. The forming plasmin generated *p*-nitroaniline, the absorbance of which was continuously recorded at 405 nm (A_405_) with a Zenith 200rt spectrophotometer (Anthos Labtec Instruments GmbH, Salzburg, Austria). The measured values were plotted vs. time squared (*t*^*2*^) yielding a linear relationship according to the equation *ΔA*_*405*_ *= 0.5εk*_*1*_*k*_*cat*_*[tPA] t*^*2*^ 15, where *ε* = 12.6 mm^−1^ cm^−1^ is the extinction coefficient of p-nitroaniline 16, *k*_*1*_ = 350 min^−1^ is the turn-over number of plasmin on Spectrozyme-PL 16, *k*_*cat*_ and *[tPA]* are the catalytic constant for plasminogen activation and the concentration of tPA in the reactive layer on the surface of fibrin, respectively 17. The term *V*_*app*_ *= k*_*cat*_*[tPA]* is equivalent to the apparent maximal rate of plasminogen activation in the reactive layer of fibrin and was determined from linear regression according to the abovementioned equation (Curve fitting toolbox v. 3.2.1 of Matlab 2012a).

### Evaluation of fibrin rigidity

Fibrinogen at 7.4 μm in 10 mm HEPES pH 7.4 buffer containing 150 mm NaCl and the investigated ECM components at final concentrations of 50 nm for aorta decorin and decorin core protein or 50 μg mL^−1^ for collagen fragments, dermatan sulfate and chondroitin sulfate was mixed with 10 nm thrombin and 410 μL of the clotting mixture was transferred to the stationary plate of HAAKE RheoStress 1 oscillation rheometer (Thermo Scientific) thermostatted at 37 °C. The cone (Titanium, 2° angle, 35 mm diameter) of the rheometer was brought to the gap position and shear strain (γ) of 0.015 was imposed exactly at 2 min after the addition of thrombin. Measurements of storage modulus (*G'*) and loss modulus (*G''*) were taken at 1 Hz in the course of 15 min with HAAKE RheoWin data manager software v. 3.50.0012 (Thermo Scientific) 18. After this 15-min clotting phase, determination of the flow limit of the fibrin gels was performed on the same samples by increasing the applied shear stress (*τ*) from 0.01 to 500.0 Pa stepwise in 150 s and measurements of the resulting strain were used for calculation of the viscosity modulus (the critical shear stress *τ*_0_ determined by extrapolation of the fall in viscosity to 0 was used as indicator of the gel/fluid transition in the fibrin structure).

### Confocal microscopic imaging

Fibrin clots were prepared from 6 μm fibrinogen, 2% of which was Alexa Fluor® 546-conjugated fibrinogen (Invitrogen Life Technologies, Budapest, Hungary) in 10 mm HEPES buffer pH 7.4 containing 150 mm NaCl, 2 mm CaCl_2_, 1.5 μm plasminogen and the tested additives with 16 nm thrombin for 30 min at room temperature in sterile, uncoated IBIDI VI 0.4 μ-slides (Ibidi GmbH, Martinsried, Germany). Thereafter 4 μg mL^−1^ tPA-YFP (tPA with Yellow Fluorescent Protein fused to its C-terminal expressed using pFastBac-tPA as previously described) 14 was added to the edge of the clot and the fluorescence (excitation wavelength 488 nm, emission wavelength 525 nm for tPA-YFP detection and excitation wavelength 543 nm, emission wavelength 575 nm for Alexa546-fibrinogen detection) was monitored with Confocal Laser Scanning System LSM710 (Carl Zeiss GmbH) taking sequential images of the fluid-fibrin interface at a distance of approximately 50 μm from the chamber surface with identical exposures and laser intensities using a Plan-NeofluarX10/0.5 objective.

### Statistical analysis

The distribution of the data on fiber diameter was analyzed according to an algorithm used previously 19: theoretical distributions were fitted to the empirical data sets and compared using Kuiper test and Monte Carlo simulation procedures. The statistical evaluation of other experimental measurements in this study was performed with the Kolmogorov–Smirnov test (Statistics Toolbox 8.0 of Matlab R2012a).

## Results

**Fig. 1:**
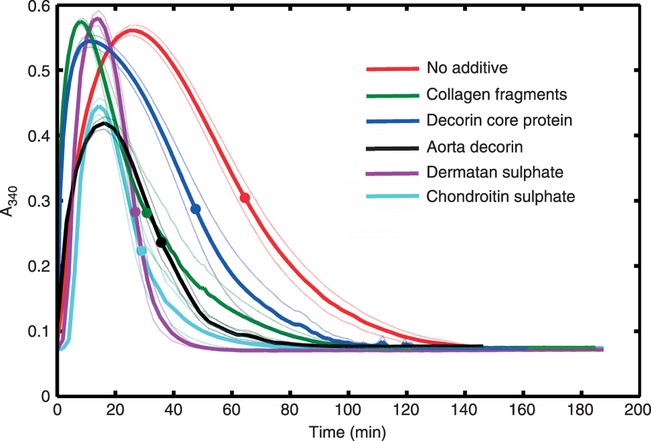
Fibrin clot lysis assay. Fibrin clots containing plasmin and the indicated additives at final concentrations 50 μg mL^−1^ for collagen fragments, dermatan sulfate or chondroitin sulfate and 50 nm for decorin core protein or aorta decorin were prepared and the absorbance was continuously measured at 340 nm (turbidity, A_340_). Mean values of eight measurements from three independent experiments (continuous lines) and standard deviation (SD) values above and below mean (dotted lines) are shown. Symbols indicate half-maximal values of A_340_ reported as lysis time in Table 1.

To model the impact of ECM components on the lytic susceptibility of fibrin formed in the immediate vicinity of proteolytically modified blood vessel walls, composite clots were prepared from fibrin and purified proteoglycans or their fragments. In this experimental setting, when clotting and fibrinolysis were initiated simultaneously, the time course of fibrin formation and dissolution reflects the global stability of fibrin clots exposed to plasmin, the major fibrinolytic enzyme. The presence of ECM proteins or glycosaminoglycans at concentration corresponding to only several per cent of the scaffold-forming fibrin concentration resulted in a significantly less stable clot structure, reflected in the shorter lysis time of the composite clots (Fig. 1 and Table 1). The presence of a sugar subunit in the structure of the modifier enhances the lytic susceptibility of the clots, as demonstrated by the difference in the lysis time for clots with full and core decorin, whereas the strongest destabilizing effects were related to the highly negatively charged glycosaminoglycans (chondroitin sulfate and dermatan sulfate). Noteworthy differences were seen in the ascending phase of the turbidity curves in Fig. 1 indicating accelerated formation of the composite clots containing proteins (shorter time to reach half-maximal turbidity in the presence of collagen fragments, glycosylated or core decorin, Table 1), whereas the isolated glycosaminoglycans either had no effect (dermatan sulfate) or delayed clot formation (chondroitin sulfate). Because a potential mechanism of the observed enhancement in the formation and lysis of the fibrin clots could be a direct effect on the activity of the enzymes involved (thrombin and plasmin), their activity was measured in a fibrin-free system on small peptide substrates for thrombin and plasmin (Spectrozyme-TH and Spectrozyme-PL, respectively). None of the investigated ECM components applied at the concentrations as in Fig. 1 influenced the enzyme activities in the absence of fibrin (data not shown).

**Table 1:** Clotting and lysis parameters of composite fibrin clots

Additive	None	fDCN	cDCN	CF	DS	CS
Clotting time (min)	5.6 ± 0.3	2.9 ± 0.1*	1.1 ± 0.1*	0.9 ± 0.1*	5.4 ± 0.1	7.1 ± 0.3*
Lysis time (min)	63.9 ± 8.7	34.9 ± 4.4*	47.6 ± 8.9*	30.4 ± 12.2*	25.8 ± 3.1*	28.0 ± 6.4*

Clots containing 6 μm fibrin, 6 nm plasmin and the indicated additives were prepared and the values of the clotting and lysis time were determined as the time needed to reach half-maximal turbidity on the ascending and descending part of the turbidity curves illustrated in Fig. 1. The values are reported as mean and standard deviation of at least eight measurements from three independent experiments. fDCN, 50 nm aorta decorin; cDCN, 50 nm decorin core protein; CF, 50 μg mL^−1^ collagen fragments; DS, 50 μg mL^−1^ dermatan sulfate; CS, 50 μg mL^−1^ chondroitin sulfate.

* *P* < 0.05 according to the Kolmogorov–Smirnov test in comparison to pure fibrin.

**Fig. 2:**
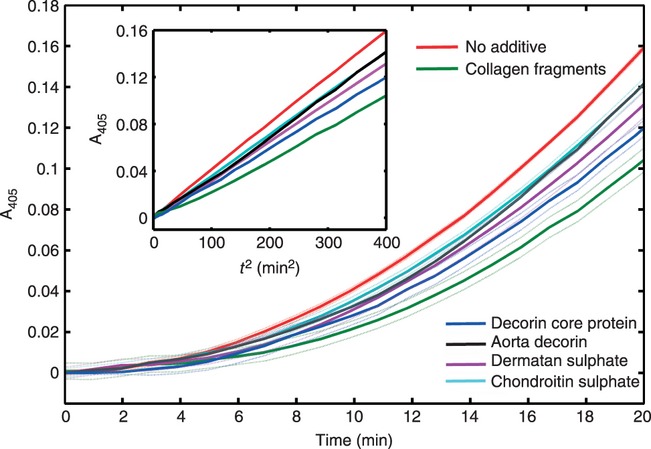
Plasminogen activation on the surface of fibrin. Fibrin clots containing plasminogen and the indicated additives were prepared and after the addition of tissue plasminogen activator (tPA) and the plasmin substrate Spectrozyme-PL the absorbance was continuously measured at 405 nm (A_405_). Mean values of eight measurements from three independent experiments (continuous lines) and standard deviation (SD) values above and below the mean (dotted lines) are shown. Inset: Secondary plots of the raw data that are used for the calculation of the apparent maximal activation rates (*V*_*app*_) reported in Table 2 (*ΔA*_*405*_*=0.5εk*_*1*_*V*_*app*_*t*^*2*^, for symbols see ‘Materials and methods’).

**Fig. 3:**
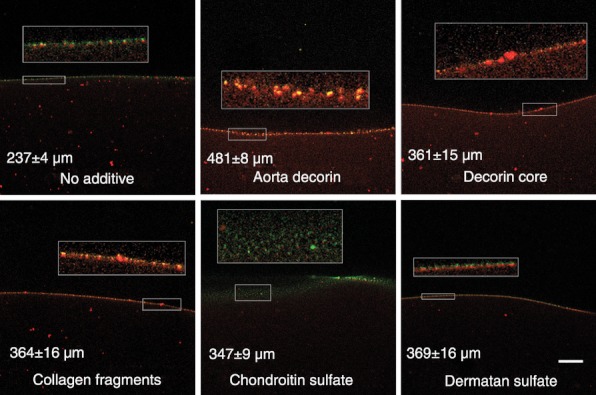
Penetration of tissue plasminogen activator (tPA)-YFP into fibrin in the course of lysis. Clots were prepared from fibrinogen containing Alexa^546^-labeled fibrinogen, plasminogen and the indicated additives at final concentrations 50 μg mL^−1^ for collagen fragments, dermatan sulfate or chondroitin sulfate and 50 nm for decorin core protein or aorta decorin. After 30-min clotting tPA-YFP was added to fibrin and the fluid/fibrin interface was monitored by confocal laser scanning microscopy using double fluorescent tracing (excitation 488 nm/emission 525 nm for tPA-YFP and excitation 543 nm/emission 575 nm for fibrin). Images are shown for the 25th min after the application of tPA-YFP, scale bar = 100 μm. The tPA-related fluorescence stains in green, whereas the fibrin is shown in red in these images and selected regions are digitally magnified four-fold to illustrate the structural pattern of the interface layer. At 0 time the edge of the fibrin clot was approximately at the same position near the top of each field of observation. The numbers in each panel indicate the distance for penetration of tPA-YFP in the clot at 25 min (mean and standard deviation from three samples, *P* < 0.05 for all additives according to the Kolmogorov–Smirnov test in comparison to pure fibrin).

In the experimental setup used in Fig. 1, plasmin activity is detected on the same substrate (fibrin) that serves as a cofactor in tPA-dependent plasminogen activation. Thus any effects of changes in fibrin structure on the rates of zymogen activation are complicated by changes in the susceptibility of modified fibrin as a substrate for plasmin. In order to identify the isolated effects on plasminogen activation, plasmin generated on the surface of fibrin was measured using a small peptide substrate (Spectrozyme-PL) that can efficiently compete with fibrin and prevents the digestion of the cofactor (Fig. 2). The apparent maximal plasminogen activation rate (*V*_*app*_) decreased to a statistically significant degree in the presence of ECM components; 12–29% reduction in *V*_*app*_ could be observed (Table 2). This modest attenuation of plasminogen activation on the surface of fibrin reflects either decreased catalytic turnover (*k*_*cat*_) or impaired interfacial penetration (*[tPA]*) in the modified fibrin structure (*V*_*app*_ = *k*_*cat*_*[tPA]*). If plasminogen was activated in the absence of fibrin in a two-stage assay 20, the investigated modifiers did not affect the activation rate precluding direct effects on tPA (data not shown). The lack of effects in free solution and the variable inhibition of plasminogen activation on the fibrin surface pointed to a role for the modification of fibrin structure in the reactive interface layer that is known to profoundly affect both tPA and plasminogen binding to fibrin, an essential determinant of plasmin generation in fibrin clots 14,21. The dynamics of this process was addressed with confocal microscopic studies using fluorescent tPA-YFP (Fig. 3). In all clots, tPA-YFP accumulated in a sharp interfacial layer, which moved as a result of fibrin lysis by the generated plasmin. The rate of migration of this front (Table 2) correlated with the rates of plasmin-mediated fibrin lysis (Fig. 1) rather than with the rate of overall plasmin generation (Fig. 2). The spatial distribution of the fluorescent tPA in the rearranging fibrin structure provides a mechanistic explanation for this effect. One of the modifiers, chondroitin sulfate, resulted in a broader homogenous tPA-rich band in fibrin, whereas in the presence of the other additives tPA co-localized with aggregates of partially degraded fibrin (the coincident green, tPA and red fibrin fluorescence is seen as yellow grains in Fig. 3). A similar granular pattern of tPA accumulation coupled to faster lysis, in spite of slower plasminogen activation has been previously reported for fibrin composed of thick fibers 14, suggesting a significant role for the structure of the fibrin network in these composite clots.

**Table 2:** Plasminogen activation on the surface of composite fibrin clots

Additive	None	fDCN	cDCN	CF	DS	CS
*V*_*app*_ for Pn formation (nm min^−1^)	0.18 ± 0.007	0.160 ± 0.011*	0.139 ± 0.008*	0.130 ± 0.015*	0.150 ± 0.015*	0.161 ± 0.011*
Relative activation rate of Pg	1	0.87	0.76	0.71	0.82	0.88
Relative migration rate of tPA	1	2.03	1.52	1.54	1.46	1.56

tPA and plasmin substrate Spectrozyme-PL were added to clots containing 3 μm fibrin, 0.1 μm plasminogen and the indicated additives and the values of the apparent activation rate (*V*_*app*_) were determined as the slopes of the plots illustrated in the Inset of Fig. 2. The values are reported as mean and standard deviation of at least eight measurements from three independent experiments. The relative migration rate of tPA in lysing fibrin has been calculated from the position of the tPA-YFP-rich layer on the surface of fibrin clots as illustrated in Fig. 3. fDCN, 50 nm glycosylated aorta decorin; cDCN, 50 nm decorin core protein; CF, 50 μg mL^−1^ collagen fragments; DS, 50 μg mL^−1^ dermatan sulfate; CS, 50 μg mL^−1^ chondroitin sulfate.

* *P* < 0.05 according to the Kolmogorov–Smirnov test in comparison to pure fibrin.

**Fig. 4:**
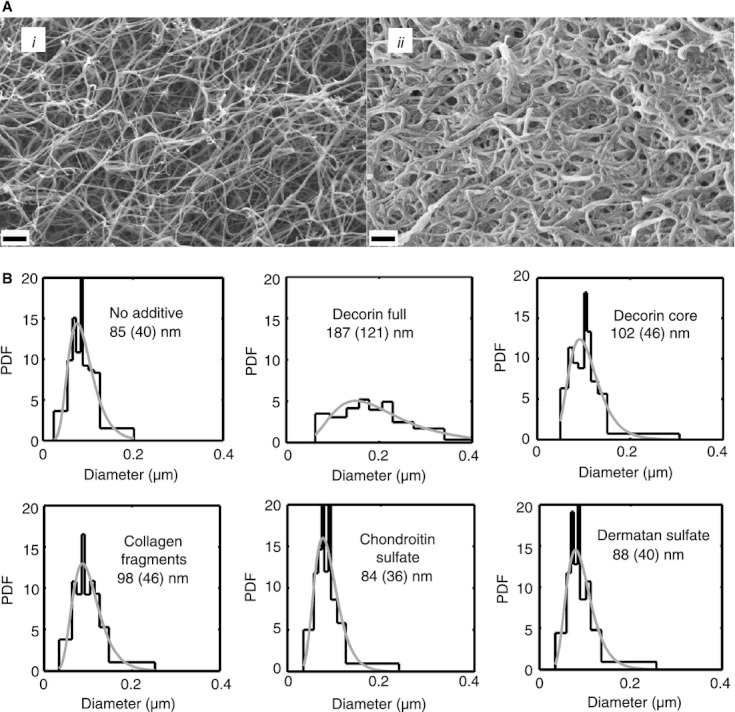
Ultrastructure of the fibrin network. (A) Fibrinogen (4.8 μm) without any additive (i) or containing 50 nm fully glycosylated decorin; and (ii) was mixed with thrombin and after 1 h clots were fixed with glutaraldehyde, dehydrated and examined with scanning electron microscopy (SEM). Scale bar = 1 μm. (B) Morphometric analysis of fibrin structure. Fibrin clots containing the indicated additives were prepared and SEM images taken as illustrated in panel A. The diameter of 300 fibers was measured and their empiric (black histograms) as well as best-fitted theoretical (gray curves) probability density function (PDF) was determined. Median values and the interquartile range are shown for the theoretical distributions of the diameter values.

Further structural details underpinning the observed changes in the enzymatic susceptibility and the tPA-cofactor function of the composite fibrin matrices were investigated using SEM (Fig. 4A). Morphometric analysis of the SEM images indicated a significant increase in fiber diameter in all cases when fibrin was polymerized in the presence of protein additives (decorin or collagen fragments). Although the negatively charged chondroitin sulfate and dermatan sulfate did not significantly influence the architecture of the fibrin network individually, fully glycosylated decorin, containing the same sugar subunits, caused the most pronounced modification of fibrin structure (Fig. 4B).

**Fig. 5:**
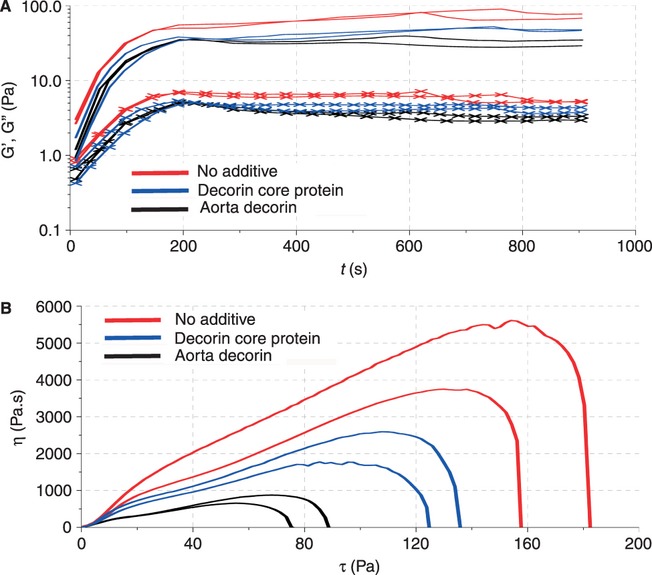
Modification of the viscoelastic properties of fibrin by extracellular matrix components. (A) Fibrinogen (7.4 μm) containing 50 nm decorin (fully glycosylated aorta or core protein) was mixed with thrombin and the storage modulus (G', continuous line) and the loss modulus (G'', crossed line) were measured using an oscillation rheometer. (B) After 15-min clotting, stepwise increasing shear stress *τ* was applied to fibrin formed in the gap space of the oscillation rheometer and viscosity (*η*) was measured. The critical values *τ*_*0*_, which define the gel/fluid transition of fibrin, are shown in Table 3.

The viscoelastic properties of the composite fibrin clots were approached using rheological methods (Fig. 5). Fully glycosylated decorin exerted the most pronounced effects on the rigidity of fibrin (decrease in the storage modulus *G'* by about 40%) and its susceptibility to structural rearrangements upon deformation (decrease in the loss modulus *G''* by about 25%) (Table 3). All other modifiers had no effect on *G'* and *G''* values (except for a moderate decrease in *G'* related to collagen fragments). The only parameter that was significantly decreased by all ECM components (except the core protein of decorin) was the critical shear stress (*τ*_0_) causing an abrupt decline in viscosity of fibrin (gel/fluid transition) (Table 3).

**Table 3:** Viscoelasticity parameters of composite fibrin clots

Additive	None	fDCN	cDCN	CF	DS	CS
*G'* (Pa)	54.29 ± 12.98	33.20 ± 3.68*	47.04 ± 0.62	40.70 ± 2.88*	41.13 ± 10.68	47.81 ± 9.20
*G''* (Pa)	4.58 ± 0.90	3.38 ± 0.50*	4.07 ± 0.43	3.83 ± 0.38	3.77 ± 0.65	4.45 ± 0.74
*G''/G'*	0.086 ± 0.015	0.102 ± 0.006*	0.086 ± 0.008	0.094 ± 0.008	0.093 ± 0.007	0.094 ± 0.006
*τ*_*0*_ (Pa)	154.42 ± 24.34	85.58 ± 10.65*	130.25 ± 7.85	95.56 ± 9.40*	103.89 ± 8.50*	111.01 ± 15.81*

Clots containing 7.3 μm fibrin and the indicated additives were prepared and their rigidity was evaluated in an oscillation rheometer. The values of the storage modulus (G'), the loss modulus (G'') and the loss tangent (tanδ = *G''/G'*) are shown for the 15th min of clotting, when they reached a plateau, whereas the critical shear stress *τ*_0_ was determined by extrapolation of the fall in viscosity to 0 as illustrated in Fig. 5B. Mean and standard deviation of at least three measurements are shown. fDCN, 50 nm glycosylated aorta decorin; cDCN, 50 nm decorin core protein; CF, 50 μg mL^−1^ collagen fragments; DS, 50 μg mL^−1^ dermatan sulfate; CS, 50 μg mL^−1^ chondroitin sulfate.

* *P* < 0.05 according to the Kolmogorov–Smirnov test in comparison to pure fibrin.

**Fig. 6:**
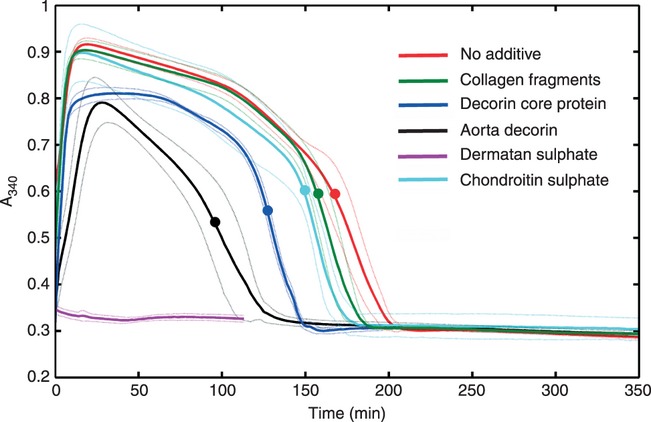
Plasma clot lysis. Citrated human plasma was supplemented with 50 ng mL^−1^ tissue plasminogen activator (tPA) and the indicated additives at final concentrations 50 μg mL^−1^ for collagen fragments, dermatan sulfate or chondroitin sulfate and 50 nm for decorin core protein or aorta decorin. Clotting was initiated with CaCl_2_ and thrombin at 12.5 mm and 15 nm final concentrations, respectively, and the absorbance was continuously measured at 340 nm (turbidity, A_340_). Mean values of eight measurements from three independent experiments (continuous lines) and standard deviation (SD) values above and below the mean (dotted lines) are shown. Symbols indicate half-maximal values of A_340_, *P* < 0.05 for all additives according to the Kolmogorov–Smirnov test in comparison to additive-free clot lysis time.

In order to evaluate the validity of the observed effects in a more physiological environment, tPA-mediated lysis of blood plasma clots was examined (Fig. 6). Dermatan sulfate (at 50 μg mL^−1^) completely blocked the formation of plasma clots in line with its known potentiating effect on the inactivation of thrombin by heparin cofactor II 22–24. Plasma clots containing all other components lysed at faster rates following the trend of plasmin-mediated (Fig. 1) and tPA-mediated (Fig. 3) fibrin lysis.

## Discussion

The *in vitro* models applied in this study address the question of lytic susceptibility and mechanical stability of fibrin structures formed when products of limited proteolysis are released from the arterial wall and coincide with fibrin polymerization. The concentrations of fibrinogen and the ionic environment used in the present studies correspond to the *in vivo* conditions found in blood plasma, whereas the vascular wall components (proteins and their fragments or glycosaminoglycans) were present in amounts up to 2.5% by mass of the amount of fibrin. Thus, the effects reported here require minimal exposure of ECM components at the site of thrombus formation, and the concentrations used are reasonable in view of the massive collagen and decorin deposits observed in primary atherosclerotic, as well as in restenotic lesions after coronary angioplasty 25 and the extensive proteolytic modification of the vascular wall induced by leukocytes 5. At present, in spite of qualitative evidence for significant abundance in atherosclerotic lesions of the ECM components investigated in the current study, there are scarce quantitative data on their concentrations. However, the example of chondroitin sulfate, which is present at 2 μg mL^−1^ basal concentration in plasma, but at 2-fold higher concentration when platelets are activated 26, suggests the local concentration could be orders of magnitude higher, before dilution in several liters of circulating blood.

The mechanical stability of fibrin structures that form efficient hemostatic plugs at sites of vessel injury is definitely compromised if ECM components co-polymerize. Significantly lower shear stress is needed to liquefy a fibrin gel in the presence of glycosylated decorin, collagen fragments, dermatan sulfate or chondroitin sulfate (*τ*_0_ in Table 3). The strongest rheological effects are produced by fully glycosylated decorin, which also causes the most pronounced changes in fibrin structure (Fig. 4). In agreement with earlier reports 18, thicker fibrin fibers were coupled to increases in the loss tangent parameter (tanδ = *G''/G'*) (Table 3). According to the interpretation of rheological parameters suggested in Ref. 18, the loss tangent reflects the dissipation of energy originating from molecular rearrangements in the fibrin network caused by mechanical forces (dissociation of monomers within protofibrils or of protofibrils within fibers). This parameter indicates that in spite of the identical trend of changes (decrease) in the rigidity (storage modulus *G'*) and dissipation energy (loss modulus *G''*) the fibrin structure softened by aorta decorin is relatively more susceptible to rearrangements of fibrin monomers or protofibrils than pure homogeneous fibrin. Rheology studies were performed in the absence of CaCl_2_, because our fibrinogen preparation contained factor FXIII and, as also shown by others 18, in the presence of physiological concentrations of CaCl_2_ FXIIIa-dependent cross-linking continues for several hours. Thus, the G', G'' values do not reach a plateau by 2 h, in contrast to the situation seen without CaCl_2_ (Fig. 5). In order to obtain reliable estimates for parameters of the mechanical properties of the clots we chose to perform the rheology measurements in the absence of CaCl_2_ and FXIIIa-dependent cross-linking. As other techniques used in these studies (light absorbance in turbidimetry, fluorescence intensity in confocal microscopy, fiber diameter in scanning electron microscopy) were not sensitive to cross-linking, physiological concentrations of CaCl_2_ were included in these systems. In view of recent data that the antifibrinolytic function of FXIII is exclusively expressed through α_2_-plasmin inhibitor cross-linking 27, the similar pattern of results observed in assay systems that contain FXIIIa, but no α_2_-plasmin inhibitor (fibrin clots in Figs. 1 and 3) or both FXIIIa and α_2_-plasmin inhibitor (plasma clots in Fig. 6) suggests that the reported ECM effects are independent of the function of FXIII in fibrinolysis.

Functional assays were investigated that could discriminate effects in the two stages of the classic fibrinolytic pathway: plasminogen activation by tPA on a fibrin template and fibrin dissolution by plasmin (reviewed in 28). When plasminogen was activated by tPA applied to the surface of fibrin clots (Fig. 2), a low-degree inhibition of activation was observed if clots contained various components derived from the vessel wall. MMP-8-digested collagen fragments and decorin core protein resulted in the strongest decrease in the apparent maximal rate of activation by 25–30% (Table 2), whereas the presence of glycosaminoglycans (either in free form or attached to decorin) moderated this effect to 12–18% inhibition. Because none of the investigated modifiers affected plasminogen activation in the absence of fibrin (data not shown), the observed effects could be explained by modulation of the interfacial phenomena on the surface of the composite clots. According to our earlier data from identical activation assays 14, thicker fibers are coupled to impaired cofactor function of fibrin in tPA-dependent plasminogen activation. In agreement with this general trend, in the present study, we observed the strongest inhibition of plasminogen activation in fibrin matrices containing collagen fragments and decorin core protein, which caused significant increases in the fiber diameter (Fig. 4). However, the thickest fibers seen in the presence of fully glycosylated aorta decorin produced less pronounced inhibition, which is probably related to independent effects of the negatively charged glycosaminoglycan side chains resulting in enhanced penetration or efficient clustering of tPA in the interfacial reactive layer of fibrin. Our data derived from the distribution of fluorescent tPA in the interfacial layer of lysing fibrin (Fig. 3) provides evidence that both of these hypothetic mechanisms are operational; the former in clots containing chondroitin sulfate, the latter with the other investigated ECM components.

The second stage of the fibrinolytic process, the digestion of fibrin by plasmin, was more profoundly affected by the components of the ECM investigated in these studies (Fig. 1). In all cases the composite clots lysed faster than pure fibrin, but the increase in rate depended on the type of modifier. Decorin core protein shortened the lysis time by 25%, whereas the presence of fully glycosylated aorta decorin, collagen fragments or isolated glycosaminoglycans resulted in approximately two-fold faster lysis (Table 1). The general trend was that faster lysis was coupled to faster clotting in the presence of protein modulators, whereas glycosaminoglycans enhanced plasmin catalyzed lysis in spite of the unaltered or even delayed clotting phase. In line with our recent findings 14, and earlier work (reviewed in 6), the coarse fibrin structures (thicker fibers, Fig. 4) formed in the presence of decorin core protein, aorta decorin or collagen fragments were lysed faster. However, in the case of isolated dermatan sulfate and chondroitin sulfate no significant structural alterations could be detected in the ultrastructure of fibrin with SEM (Fig. 4) in spite of a significant enhancement in lysis (Fig. 1). It is noteworthy that the lack of an effect on the fibrin structure was accompanied by differential effects on the kinetics of clotting (Fig. 1); in contrast to the accelerated fibrin formation in the presence of protein modifiers, the same glycosaminoglycans either did not change or prolonged the clotting time (Table 1). Because we could not detect any effects on thrombin activity measured on a small synthetic chromogenic substrate (data not shown), an alternative background of the modified clotting profiles should be sought in the macromolecular nature of the fibrinogen substrate. For example, in the literature 9,29 there is some evidence for interactions of fibrinogen and ECM components that could change the kinetics of fibrin monomer formation and/or polymerization, but clarification of the exact mechanism requires further studies.

As our data demonstrate that fibrin lysis by plasmin (Fig. 1) and plasminogen activation (Fig. 2) are affected in a discordant manner by the modulators under investigation when present in the clot, the overall outcome cannot be predicted when the two stages of fibrinolysis operate simultaneously. To clarify this issue two types of assay were performed, in which clot lysis was initiated by tPA/plasminogen (Figs. 3 and 6). These data show that the effects exerted on plasmin-mediated lysis dominate the overall fibrinolytic process, which is accelerated by all examined modulators in spite of the modest inhibition of plasminogen activation. Importantly, this enhanced lytic susceptibility is preserved even at physiological levels of blood plasma inhibitors (Fig. 6).

In summary, our present study indicates that in spite of subtle differences in mechanism, vascular wall components entrapped in the structure of fibrin generally compromise the mechanical and chemical stability of fibrin, they increase its susceptibility to shear stress and to plasmin, the major fibrinolytic protease. These effects require minimal concentrations of modulators. In an earlier study 10 thinner fibers and mildly suppressed tPA-dependent fibrinolysis were reported in the presence of recombinant decorin. However, the fibrin used in that work was prepared with 20-fold lower thrombin and 2-fold higher Ca^2+^ concentrations (conditions known to profoundly affect the structure and lytic susceptibility of fibrin, 14), and the decorin concentration was 10-fold higher. In addition, the recombinant decorin used in that study was not glycosylated in contrast to the natural aorta decorin used by us and, as clearly demonstrated by the present data, some functional effects in fibrinolysis depend on the nature of the glycosaminoglycan.

Concerning the *in vivo* implications of the reported results, differences between physiological wound healing and pathological thrombi in ruptured atherosclerotic plaques should be considered. Hemostatic plugs are formed over acutely injured vessel wall when blood makes contact with largely intact (except for the localized mechanical damage) subendothelial structures. In contrast, the atherosclerotic plaque is a focus of chronic inflammation and thus, after rupture, blood is exposed to an environment that has been extensively processed by proteases and other hydrolases released from inflammatory cells. Hence, the processes investigated here are probably observed in pathological thrombi. Nevertheless, atherosclerotic plaque lesions are sites of a dynamically changing structure because of altered proteoglycan synthesis 30,31 and proteolytic destruction 3 and additional studies are required to investigate the quantity and nature of the protein and glycosaminoglycan modulators in thrombi attached to, or derived from, atherosclerotic plaques.
